# SLC52A3-Related Riboflavin Transporter Deficiency in Two Saudi Siblings: A Case Report

**DOI:** 10.7759/cureus.115131

**Published:** 2026-08-25

**Authors:** Fadi Busaleh, Nabil Almajhad, Ola H Alabbad, Dalal M Alshehri, Reham R Alhassan, Ghaliah Y Alhejji, Mariyyah A Al Jamea, Amnah F Alomran, Shomos H Aldulaim, Fatema M Almohammad Saleh, Anwar F Alqadeep

**Affiliations:** 1 Pediatrics/Pediatric Neurology, Maternity and Children's Hospital, Al-Ahsa, SAU; 2 Pediatrics/Pediatric Neurology, Maternity and Children’s Hospital, Al-Ahsa, SAU; 3 Medicine, College of Medicine, King Faisal University, Al-Ahsa, SAU; 4 Medicine, King Faisal University, Dammam, SAU; 5 Pediatrics, King Faisal University, Al-Ahsa, SAU; 6 Medicine, King Faisal University, Al-Ahsa, SAU

**Keywords:** brown-vialetto-van laere syndrome, bulbar palsy, developmental regression, sensorineural (sn) hearing loss, slc52a3

## Abstract

Brown-Vialetto-Van Laere syndrome (BVVLS), or riboflavin transporter deficiency (RTD), is a rare but treatable neurodegenerative disorder caused by pathogenic variants in the riboflavin transporter genes. We report the cases of two siblings with genetically confirmed *SLC52A3*-related RTD. The index case presented with progressive bulbar dysfunction and respiratory failure and was diagnosed after an initially challenging clinical course. During a subsequent pregnancy, prenatal genetic testing identified the same familial *SLC52A3 *variant, allowing maternal riboflavin supplementation during pregnancy and treatment of the affected sibling from birth. Following riboflavin therapy, the index case showed partial clinical improvement, while the younger sibling remained asymptomatic at one year of age. This report highlights the importance of early diagnosis and treatment of RTD and the potential benefit of starting therapy before the onset of symptoms.

## Introduction

Brown-Vialetto-Van Laere syndrome (BVVLS) is a rare neurodegenerative disorder historically characterized by progressive pontobulbar palsy, sensorineural hearing loss, and respiratory compromise. Following identification of its molecular basis, BVVLS and the clinically overlapping Fazio-Londe syndrome are now classified within the spectrum of riboflavin transporter deficiency (RTD). Most genetically confirmed cases result from biallelic pathogenic variants in *SLC52A2*, encoding riboflavin transporter 2, or *SLC52A3*, encoding riboflavin transporter 3. The terms *SLC52A2*-related RTD type 2 and *SLC52A3*-related RTD type 3 are preferable to older and inconsistently applied BVVLS type numbers [1-4].

Riboflavin is the precursor of flavin mononucleotide (FMN) and flavin adenine dinucleotide (FAD), essential cofactors for multiple cellular metabolic pathways, including mitochondrial electron transport. Defective riboflavin transport impairs energy production, particularly in tissues with high metabolic demands such as the central and peripheral nervous systems. Consequently, affected patients develop progressive neurological manifestations, including sensorineural hearing loss, bulbar dysfunction with swallowing difficulties, and respiratory muscle weakness [2-6].

Onset ranges from infancy to adulthood. Early-onset *SLC52A3*-related disease may be rapidly progressive and dominated by bulbar dysfunction and respiratory failure, while later presentations may feature hearing loss, cranial neuropathies, or sensorimotor neuropathy [3,5-7]. Untreated RTD can be fatal, whereas high-dose oral riboflavin may halt progression and produce meaningful neurological recovery. The response is greatest when treatment begins before irreversible neuronal injury [5-6,8-11].

We report the cases of two sisters with genetically confirmed *SLC52A3*-related RTD type 3 in one family and the course of the disease and its challenges.

## Case presentation

Case 1

The index case was a Saudi girl who was diagnosed with Brown-Vialetto-Van Laere syndrome at 10 months of age. She was born at term following an uncomplicated pregnancy and spontaneous vaginal delivery to first-degree consanguineous parents. There was no known family history of neuromuscular, metabolic, or hereditary disorders, and her early developmental milestones were appropriate until 9 months of age. She initially presented with progressive bulbar weakness and respiratory insufficiency.

**Figure 1:**
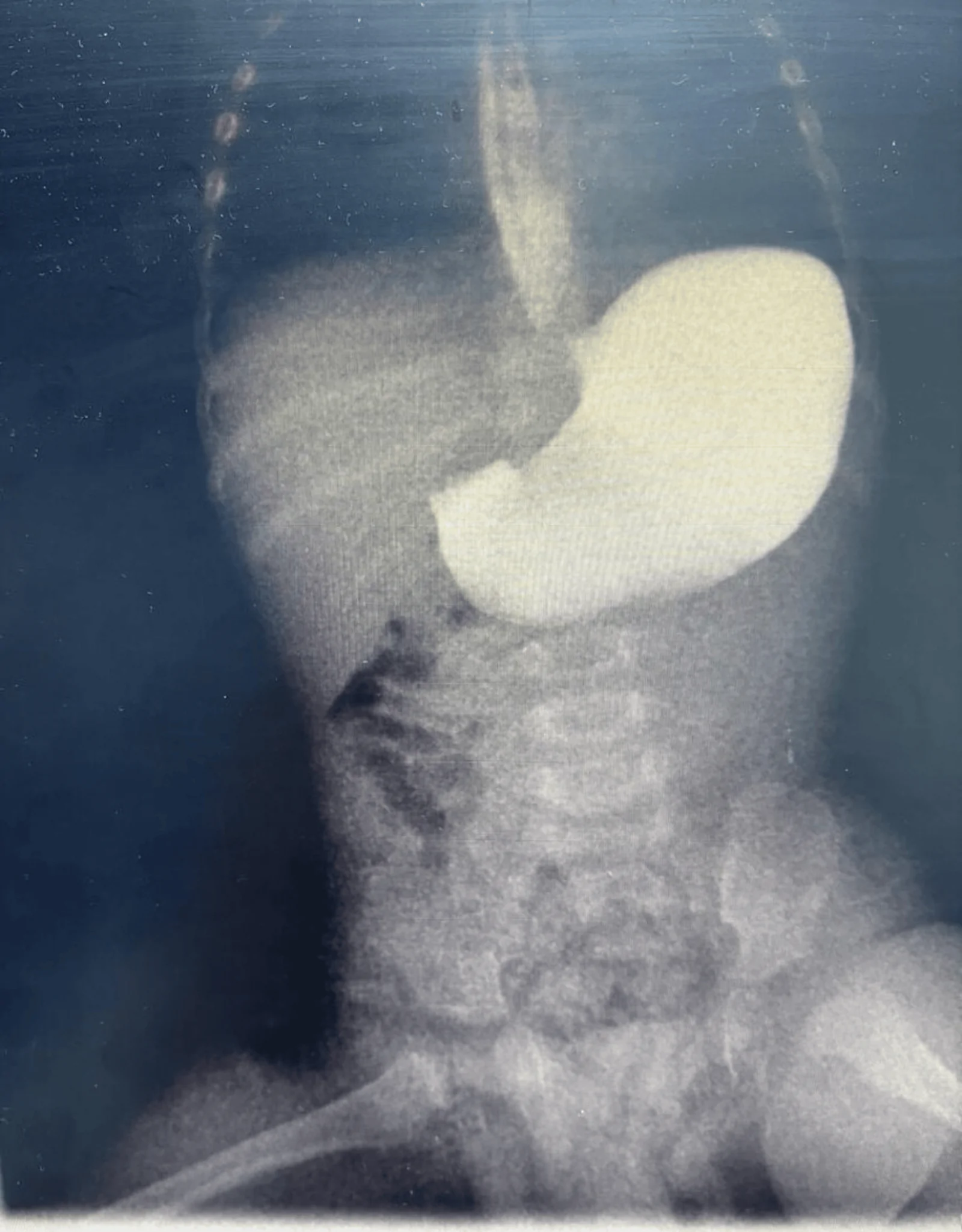
Upper gastrointestinal contrast study demonstrated Grade III gastroesophageal reflux, with reflux of contrast extending into the upper esophagus (cervical); no structural esophageal abnormality was identified.

At nine months, she developed recurrent episodes of inspiratory stridor and cyanosis following crying. Each episode reportedly lasted 10-15 minutes, and two occurred within one day, prompting hospital admission. The initial differential diagnoses included bronchiolitis, gastroesophageal reflux, and a brief resolved unexplained event (BRUE). Computed Tomography (CT) of the Brain was unremarkable. Echocardiography demonstrated a patent foramen ovale and a small secundum atrial septal defect. An upper gastrointestinal contrast study was interpreted as grade III gastroesophageal reflux (Figure 1). She improved symptomatically and was discharged on antireflux therapy.

Approximately two weeks later, she was readmitted with poor feeding, choking, decreased activity, recurrent cyanotic episodes, and type II respiratory failure requiring pediatric intensive care. Her mother then described progressive bilateral ptosis and an apparent diurnal fluctuation, with weakness becoming more pronounced toward the end of the day and improving after rest. The child had lost previously acquired abilities, including rolling, independent sitting, babbling, and attempts to stand.

On neurological examination, she was alert and had bilateral partial ptosis, reduced facial expression, excessive salivation, poor sucking, and tongue fasciculations without definite atrophy. She had generalized hypotonia and proximal-predominant weakness, and diminished deep tendon reflexes. Sensory responses to examination were preserved. There were no dysmorphic features or neurocutaneous stigmata.

**Figure 2:**
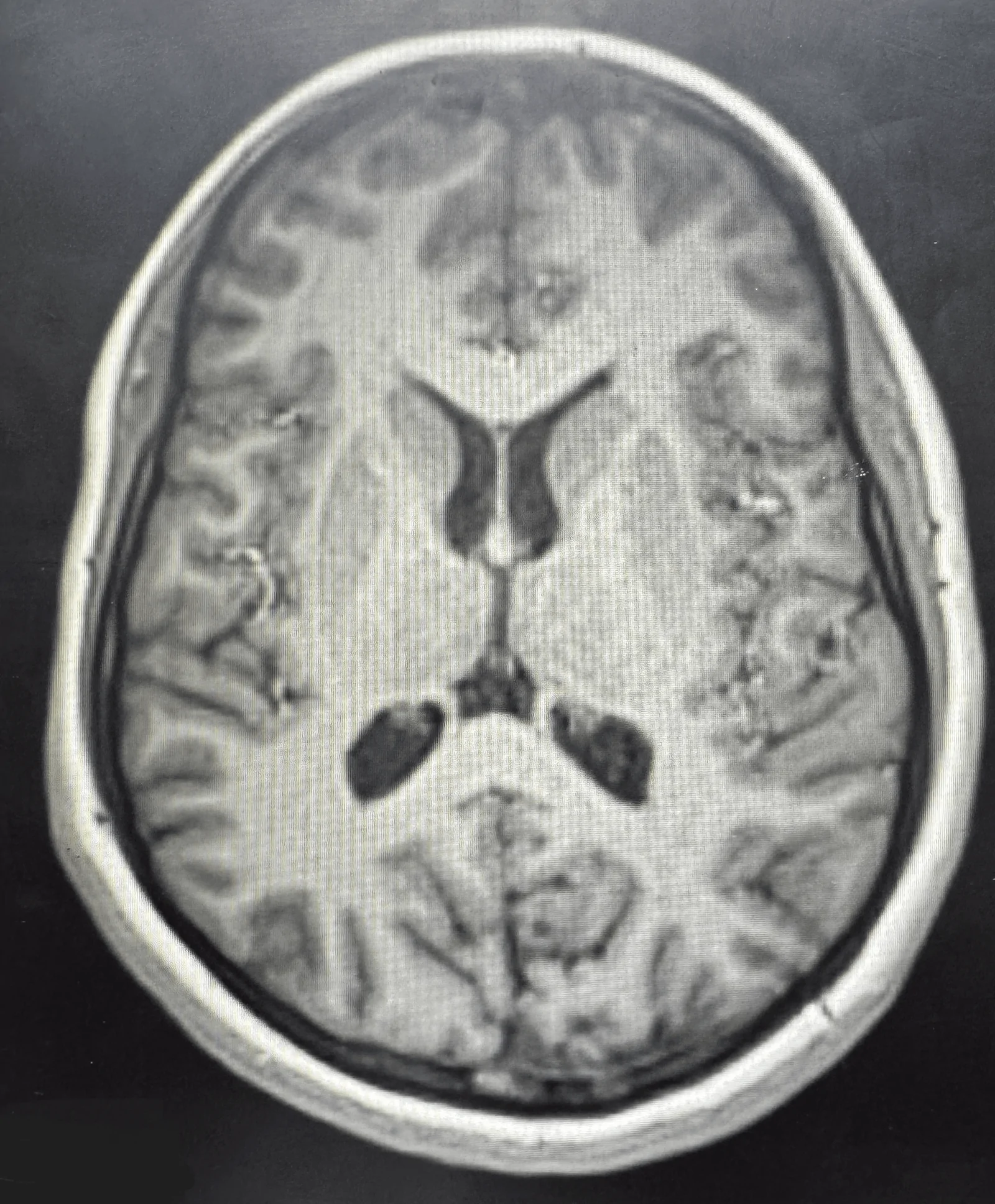
Axial T1-weighted brain MRI showing no structural brain abnormality. Gray-white matter differentiation, ventricular size, and cerebral parenchyma are preserved, with no evidence of focal lesions, cerebral atrophy, or hydrocephalus.

Routine investigations showed mild microcytic anemia and thrombocytosis, with no biochemical evidence of infection, metabolic decompensation, or a primary destructive myopathy (Table 1). Serum creatine kinase, lactate, ammonia, and lactate dehydrogenase were normal. Brain MRI was normal (Figure 2). Otoacoustic emissions were present bilaterally at that stage.

**Table 1:** Table showing initial hematological and biochemical makers

Parameter	Result	Reference range
Hematological markers		
White blood cells	13.37 × 10³/µL	6-17 × 10³/µL
Red blood cells	4.65 × 10⁶/µL	3.9–5.5 × 10⁶/µL
Hemoglobin	10.6 g/dL	≥10.5 g/dL
Hematocrit	35%	≥33%
Mean corpuscular volume	75.3 fL	≥70 fL
Mean corpuscular hemoglobin	22.8 pg	23–31 pg
Mean corpuscular hemoglobin concentration	30.3 g/dL	≥30 g/dL
Platelets	440 × 10³/µL	150-350 × 10³/µL
Biochemical and metabolic markers		
Creatine kinase	50 U/L	20-180 U/L
Lactate dehydrogenase	206 IU/L	180-430 IU/L
Ferritin	7.20 ng/mL	7-140 ng/mL
Ammonia	40.4 µmol/L	21-50 µmol/L
Lactate	1.92 mmol/L	1-3.3 mmol/L
Blood urea nitrogen	3.44 mmol/L	1.8–6.4 mmol/L
Creatinine	24.36 µmol/L	18-35 µmol/L
Sodium	141 mmol/L	135-145 mmol/L
Potassium	4.9 mmol/L	4.1-5.3 mmol/L
Chloride	97 mmol/L	97-106 mmol/L
Magnesium	1.07 mmol/L	0.63-1.05 mmol/L
Phosphate	2.30 mmol/L	1.29-2.10 mmol/L
Glucose	138 mg/dL	60-200 mg/dL

The combination of progressive bulbar dysfunction, respiratory failure, ptosis, hypotonia, developmental regression, tongue fasciculations, reduced reflexes, and normal neuroimaging raised suspicion of an inherited neuromuscular disorder. Targeted testing for *SMN1*-related spinal muscular atrophy and whole-exome sequencing were requested. Whole-exome sequencing identified a homozygous pathogenic variant in *SLC52A3*, confirming *SLC52A3*-related RTD type 3.

Oral riboflavin was started at 20 mg/kg/day. Because respiratory insufficiency and unsafe swallowing persisted, she required prolonged ventilatory support, tracheostomy, and nasogastric feeding. At the most recent follow-up, at five years of age, her motor and swallowing abilities had improved; however, her hearing deteriorated despite supplementation, and she ultimately underwent bilateral cochlear implantation at the age of 4 years. She remains under multidisciplinary respiratory, nutritional, rehabilitation, and audiological care.

Case 2

The family received comprehensive genetic counseling regarding the autosomal recessive inheritance pattern, recurrence risk, and available reproductive options. During a subsequent unplanned pregnancy, prenatal diagnosis by amniocentesis identified the same homozygous pathogenic *SLC52A3 *variant in the fetus. Following confirmation of the diagnosis, the mother was started on oral riboflavin supplementation for the remainder of the pregnancy. After delivery, the newborn continued oral riboflavin at the same maintenance dose. Genetic testing was repeated after birth and again confirmed the presence of the same homozygous pathogenic *SLC52A3 *variant.

The infant has been followed regularly through pediatric neurology and routine well-child visits. Serial developmental assessments demonstrated age-appropriate neurodevelopment, with no evidence of developmental regression, bulbar dysfunction, respiratory compromise, hearing impairment, or hypotonia. At the most recent assessment at 12 months of age, the child was able to stand while holding onto furniture, cruise along furniture, and pull to stand independently. Fine motor skills were appropriate for age, including an established pincer grasp, self-feeding with finger foods, voluntary release of objects, and transferring objects between hands. Language development was also age-appropriate, with specific use of “mama” and “baba,” response to simple verbal commands, imitation of sounds, and appropriate social interaction. 

Hearing assessment was performed twice during follow-up. Initial newborn hearing screening was normal, and repeat audiological assessment, including auditory brainstem response (ABR), remained normal with no evidence of sensorineural hearing loss. At one year of age, the genetically affected sibling remained clinically asymptomatic while continuing riboflavin therapy and regular multidisciplinary follow-up.

**Figure 3:**
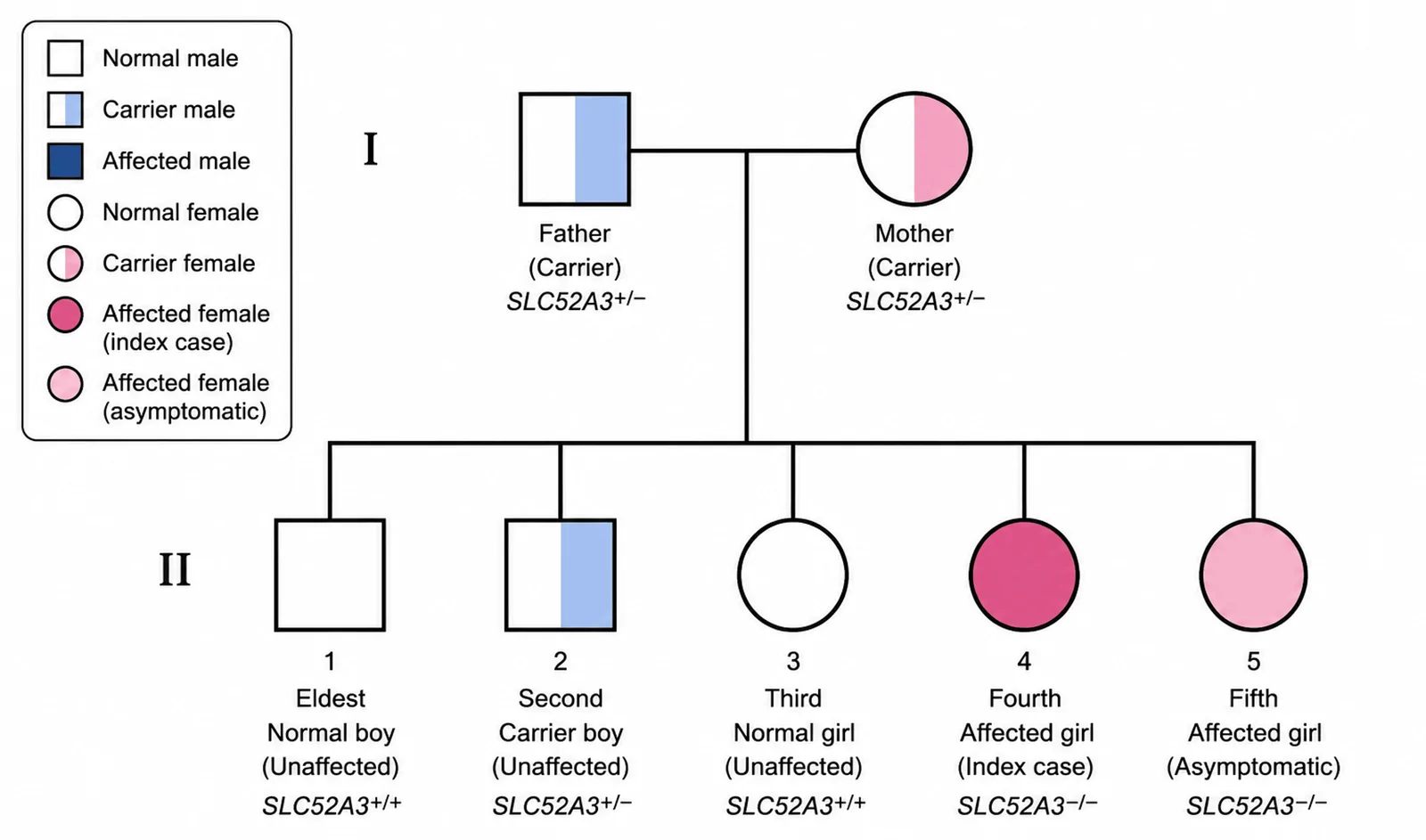
Pedigree of the family demonstrating autosomal recessive inheritance of the pathogenic SLC52A3 variant. Both parents are heterozygous carriers. Of the five siblings, one is an asymptomatic carrier, two are unaffected non-carriers, the fourth child is the symptomatic index case, and the youngest affected sibling was diagnosed prenatally and remains asymptomatic following early riboflavin therapy. Created using Microsoft Word (Microsoft Corporation, Redmond, USA).

Genetic testing was performed for all available family members. Both parents were found to be heterozygous carriers of the pathogenic *SLC52A3 *variant. The eldest son and the third child (a girl) tested negative for the familial variant. The second son was identified as a heterozygous carrier and remained asymptomatic. Both the index patient and her youngest sister were homozygous for the pathogenic *SLC52A3 *variant, confirming the diagnosis of riboflavin transporter deficiency (Figure 3).

## Discussion

Brown-Vialetto-Van Laere syndrome (BVVLS) is a rare but treatable neurodegenerative disorder caused by pathogenic variants in the *SLC52A3 *gene, resulting in riboflavin transporter deficiency (RTD). Impaired riboflavin transport disrupts mitochondrial energy metabolism, predominantly affecting the cranial and peripheral motor neurons. Consequently, patients develop progressive bulbar dysfunction, dysphagia, respiratory muscle weakness, sensorineural hearing loss, and limb weakness, with a highly variable clinical presentation [2-6].

The early presentation in our first patient (Case 1) was diagnostically challenging. Stridor and cyanotic episodes after crying were initially attributed to common pediatric conditions, including bronchiolitis and gastroesophageal reflux. The term brief resolved unexplained event (BRUE) was also considered. However, the reported duration of 10-15 minutes exceeds the usual BRUE definition, which describes a sudden, brief, resolved event in an infant younger than one year and generally lasting less than one minute [12]. Retrospectively, these episodes were better characterized as BRUE-like cyanotic events caused by evolving bulbar and respiratory neuromuscular dysfunction. The later appearance of choking, poor sucking, excessive salivation, ptosis, tongue fasciculations, hypotonia, milestone loss, and hypercapnic respiratory failure clarified the presence of combined cranial motor and lower motor neuron involvement [3-6]. Although echocardiography demonstrated a patent foramen ovale and a small secundum atrial septal defect, these findings were considered incidental and hemodynamically insignificant. There were no clinical features of heart failure, and the episodes were not consistently related to feeding or exertion, making a cardiac cause of the cyanotic events unlikely.

Respiratory compromise in RTD may result from diaphragmatic and intercostal weakness, lower motor neuron degeneration, vocal-cord dysfunction, aspiration related to bulbar weakness, or a combination of these mechanisms. In infants, respiratory failure can dominate the presentation and progress rapidly. The patient's need for prolonged ventilation and tracheostomy indicates severe neurological injury established before diagnosis. Improvement in swallowing and motor abilities after riboflavin supports biological treatment responsiveness, but persistent respiratory morbidity illustrates that supplementation cannot reliably reverse extensive motor neuron loss [5-6&8-11]. 

The reported diurnal fluctuation and ptosis initially suggested a congenital myasthenic syndrome. Congenital myasthenic syndromes may cause fatigable ptosis, bulbar weakness, stridor, episodic apnea, and respiratory crises [13-14]. However, fatigability is not exclusive to neuromuscular junction disease and may also become apparent when a child with motor neuron or peripheral nerve disease has limited physiological reserve. In the patient, tongue fasciculations, reduced reflexes, progressive milestone loss, and the combined bulbar-respiratory phenotype were more consistent with a neuronopathy than with an isolated neuromuscular transmission defect [3-5].

Spinal muscular atrophy was another important consideration because infantile hypotonia, tongue fasciculations, reduced reflexes, weakness, bulbar dysfunction, and respiratory failure are characteristic features [15]. However, prominent ptosis and evolving auditory disease favored an alternative diagnosis. Congenital myopathies may also present with hypotonia, feeding difficulty, ptosis, and respiratory weakness, often with normal or mildly elevated creatine kinase, but they do not readily explain the auditory neuropathy that is well documented in BVVLS [5-7]. Mitochondrial and other metabolic diseases can produce regression, hypotonia, and respiratory failure; however, normal lactate, ammonia, MRI, and routine screening made acute metabolic decompensation less likely. Importantly, normal biochemical studies do not exclude RTD, because acylcarnitine and other metabolic abnormalities may be absent or intermittent; definitive diagnosis therefore relies on molecular analysis of the riboflavin transporter genes, particularly *SLC52A2 *and *SLC52A3 *[2-3,5].

Fazio-Londe syndrome is a particularly relevant historical differential diagnosis. It is characterized by childhood-onset progressive pontobulbar palsy and respiratory dysfunction without the deafness classically associated with BVVLS. Molecular studies have demonstrated substantial genetic and phenotypic overlap, especially in *SLC52A3*-related disease [2-4]. The distinction based solely on hearing status is therefore unreliable early in the disease course. The patient initially had present otoacoustic emissions but later developed severe sensorineural hearing loss, illustrating how an early Fazio-Londe-like presentation may evolve into the broader BVVLS phenotype [3,5].

Present otoacoustic emissions should not be interpreted as proof of normal auditory pathway function. Otoacoustic emissions primarily reflect cochlear outer hair cell integrity and may remain present in auditory neuropathy, in which neural transmission from the inner hair cells, auditory nerve, or brainstem pathways is impaired [7,16]. Auditory neuropathy is well documented in BVVLS, and auditory brainstem response testing is therefore important even when otoacoustic emissions are preserved [7,16]. Serial surveillance is also necessary because auditory involvement may emerge after bulbar or respiratory symptoms [5,7]. This occurred in the patient, whose hearing progressed to severe loss requiring bilateral cochlear implantation. Cochlear implantation can improve auditory access and speech perception in selected patients, although outcomes vary because the pathological site may be postsynaptic or neural [7,16-18].

A central question in this case is whether developmental regression can reverse after riboflavin supplementation. Available evidence indicates that substantial improvement is possible, but recovery depends on treatment timing and the extent of irreversible neuronal injury [5,8-11]. Anand et al. reported a child who rapidly lost motor abilities and required ventilatory support but, after high-dose riboflavin, regained previous gross motor function and walked independently [8]. Across 70 molecularly confirmed cases reviewed by Jaeger and Bosch, untreated patients experienced continued progression, whereas most treated patients stabilized or improved; mortality among untreated patients was largely related to respiratory insufficiency [5]. These findings suggest that some regression reflects reversible metabolic dysfunction in surviving neurons, whereas fixed deficits reflect established neuronal loss. In our proband, improvement in motor and swallowing function after treatment suggests partial reversal, but persistent respiratory morbidity and progressive hearing loss indicate incomplete rescue after delayed diagnosis [5,8-11].

The patient's course can be compared with the recent regional cohort reported by Al Shamsi et al., the largest *SLC52A3*-related BVVLS series from the Arabian Peninsula, which included 23 patients managed at tertiary centers in Oman and Saudi Arabia [19]. Thirteen patients were symptomatic, and 10 were identified presymptomatically. Symptomatic manifestations included facial weakness, dysarthria, hearing loss, and vocal-cord paresis. Most treated symptomatic patients improved substantially but retained variable residual deficits, whereas nine of 10 presymptomatically treated patients remained asymptomatic [19]. Compared with that cohort, our proband had a particularly severe infantile respiratory phenotype requiring tracheostomy and prolonged ventilatory support. However, her partial recovery after treatment and the favorable course of her younger sibling parallel the regional observation that established disease may improve without fully resolving, whereas presymptomatic treatment is associated with markedly better outcomes [19].

Recent studies advocate initiating empirical high-dose riboflavin therapy in patients with a strong clinical suspicion of RTD, even before molecular confirmation, as diagnostic delays may result in irreversible neurological damage [10,11]. Reported therapeutic doses range from 7 to 60 mg/kg/day, with no universally accepted optimal regimen [5,10]. The patient received 20 mg/kg/day, which falls within the recommended therapeutic range. Treatment should be individualized according to clinical response and tolerability, with regular assessment of respiratory, bulbar, motor, visual, and auditory function [5,10-11].

The second patient (Case 2), sibling of the first patient (Case 1), was diagnosed prenatally after the familial *SLC52A3 *mutation had been identified in the proband. Maternal riboflavin supplementation was started during pregnancy and continued after birth. At one year of follow-up, the child remains asymptomatic. Similar outcomes have been reported by Al Shamsi et al. in presymptomatically treated patients and by Elks et al. following antenatal riboflavin therapy [19,20]. Our findings further support family screening, prenatal diagnosis, and early riboflavin treatment in at-risk families.

## Conclusions

The differential diagnosis of bulbar palsy associated with acute neurological deterioration and developmental regression in infancy is extensive. Nevertheless, early and progressive bulbar dysfunction should raise suspicion for riboflavin transporter deficiency, a potentially treatable disorder, even in the absence of the classical phenotype. Early recognition, appropriate genetic testing, and timely initiation of riboflavin therapy are critical to optimize neurological outcomes.
